# Canadian Conference on Medical Education April 13-16, 2019 Niagara Falls, Ontario, Canada

**Published:** 2019-04-03

## CONTENTS

**Introduction**

**Workshop Abstracts**

**Oral Abstracts**

**Dedicated Poster Abstracts**

**Poster Group Abstracts**

**Disclaimer:** This abstract book has been produced using author-supplied copy. Editing has been restricted to some corrections of spelling and style where appropriate. No responsibility is assumed for any claims, instructions, or methods contained in the abstracts: it is recommended that these are verified independently.

